# Image-Guided Superficial Radiation Therapy for Squamous Cell Carcinoma In Situ of the Nasal Ala in a High-Risk Surgical Candidate: A Case Report

**DOI:** 10.7759/cureus.110796

**Published:** 2026-06-13

**Authors:** Caitlin Wenske, Mahnoor Mukarram, Carlos Gomez-Meade

**Affiliations:** 1 School of Medicine, Oklahoma State University College of Osteopathic Medicine, Tulsa, USA; 2 Transitional Year Program, Abrazo Community Health Network, Goodyear, USA; 3 Dermatology, Cornerstone Skin Surgery Center, Tulsa, USA

**Keywords:** nasal ala, nonmelanoma skin cancer, nonsurgical management, poor surgical candidate, squamous cell carcinoma in situ, superficial radiation therapy, ultrasound-guided

## Abstract

Nonmelanoma skin cancers (NMSCs), including squamous cell carcinoma in situ (SCCIS), are common and considered curable with early intervention; however, management must be individualized based on tumor characteristics and patient-specific factors. While surgical approaches such as Mohs micrographic surgery are the standard of care for high-risk anatomical sites, nonsurgical modalities are essential for patients with significant comorbidities.

We present the case of an 80-year-old male with recurrent SCCIS of the right nasal ala whose medical history was significant for renal transplantation on chronic immunosuppressive therapy, chronic obstructive pulmonary disease (COPD) requiring supplemental oxygen, and impaired mobility, making him a poor surgical candidate for Mohs surgery or complex reconstruction. The patient elected treatment with image-guided superficial radiation therapy (IG-SRT), a noninvasive modality that incorporates high-resolution dermal ultrasound (HRDUS) for precise tumor measurement and treatment planning. Initial HRDUS demonstrated a tumor depth of 2.34 mm. He subsequently completed 20 fractions over six weeks, receiving a total dose of 5666.20 cGy. Expected radiation-induced skin changes developed during treatment, with a peak Radiation Therapy Oncology Group (RTOG) toxicity score of 2. Serial HRDUS demonstrated progressive tumor regression and no detectable residual tumor six weeks after treatment completion. The patient was pleased with the cosmetic outcome and experienced no alar distortion or airway compromise.

This case highlights IG-SRT as a feasible, tissue-sparing treatment option for recurrent SCCIS in medically complex patients who are poor surgical candidates while emphasizing the value of HRDUS for treatment planning and response monitoring.

## Introduction

Skin malignancies are broadly categorized into melanoma and nonmelanoma skin cancer (NMSC). While melanoma is less common, it is significantly more aggressive and associated with a high mortality rate. In contrast, NMSCs, such as basal cell carcinoma (BCC) and squamous cell carcinoma (SCC), are much more prevalent and considered curable with early recognition and treatment [1].

There are a variety of surgical and nonsurgical techniques utilized for the treatment of NMSCs; these include systemic and topical pharmacotherapy, cryosurgery, photodynamic therapy (PDT), radiotherapy, electrodesiccation and curettage (ED&C), surgical excision, and Mohs micrographic surgery [2]. Treatment plans should take into consideration tumor-specific characteristics while also prioritizing a patient-centered approach by integrating comorbidities, functional status, cosmetic considerations, and patient preferences to optimize management. 

Radiation therapy is an effective option for patients who are not ideal surgical candidates or who have tumors less amenable to other conservative treatments [3]. This type of therapy uses electromagnetic energy generated from X-rays or photons to cause DNA damage that leads to cell cycle arrest and apoptosis in rapidly dividing tissues [4]. Radiotherapy modalities used for skin cancers include superficial radiation therapy (SRT), electron-beam radiotherapy (EBRT), electronic brachytherapy (EBX), and image-guided superficial radiation therapy (IG-SRT). Among these, IG-SRT has emerged as a refined modality that incorporates real-time imaging for improved precision. Although Mohs micrographic surgery remains the standard treatment for many high-risk facial NMSCs, IG-SRT may be a valuable alternative in select patients who are poor surgical candidates or who wish to avoid surgery due to functional or cosmetic concerns.

IG-SRT is distinguished by its use of high-resolution dermal ultrasound (HRDUS) imaging to measure tumor size and depth, enabling more precise radiation dose planning, minimizing unnecessary exposure, and facilitating ongoing assessment of treatment response [5]. In contrast, the other modalities lack integrated real-time imaging for depth assessment and deliver higher energy doses without the same level of spatial precision [4]. The most common protocol utilized for IG-SRT involves three to four fractions per week at a dose of 265-280 centigray (cGy) [6]. This treatment is useful for superficial cutaneous tumors that are less than 3 mm in depth, including early-stage SCC and BCC [6]. Doses ≥300 cGy carry an increased risk of acute and chronic skin toxicity, whereas doses <265 cGy have the potential to undertreat the lesion and prolong treatment duration [6].

Squamous cell carcinoma in situ (SCCIS) represents an early form of SCC confined to the epidermis. Management can be particularly challenging when lesions arise on the nasal ala, a cosmetically and functionally sensitive site with limited tissue reserve for reconstruction. This case highlights the role of IG-SRT in the management of SCCIS of the nasal ala in an elderly patient with multiple comorbidities that made him a poor surgical candidate.

## Case presentation

An 80-year-old male with a past medical history significant for renal transplantation on chronic immunosuppressive therapy, hypertension (HTN), and chronic obstructive pulmonary disease (COPD) requiring supplemental oxygen was referred for Mohs micrographic surgery for recurrent SCCIS involving the right alar rim. The lesion had previously been treated with Mohs micrographic surgery; however, the timing of the original diagnosis and prior treatment was unavailable. The diagnosis of recurrent SCCIS had been confirmed histopathologically on biopsy prior to referral and before treatment planning for IG-SRT. His medical history was further complicated by a total hip arthroplasty, resulting in impaired ambulation and reliance on a walker.

During consultation, the lesion measured 1.8 × 1.1 cm, and the visible scar was consistent with previous biopsy and residual skin cancer. Given his significant comorbidities and potential for surgical challenges, treatment options were discussed, which included the patient having a second opinion from Otolaryngology (ENT) or head and neck surgery due to the complexity of the location and history of recurrence. ENT recommended consideration of IG-SRT due to the patient being a poor overall surgical candidate with likely need for complex and multistage reconstruction. The patient ultimately elected to proceed with radiotherapy using IG-SRT. 

**Figure 1:**
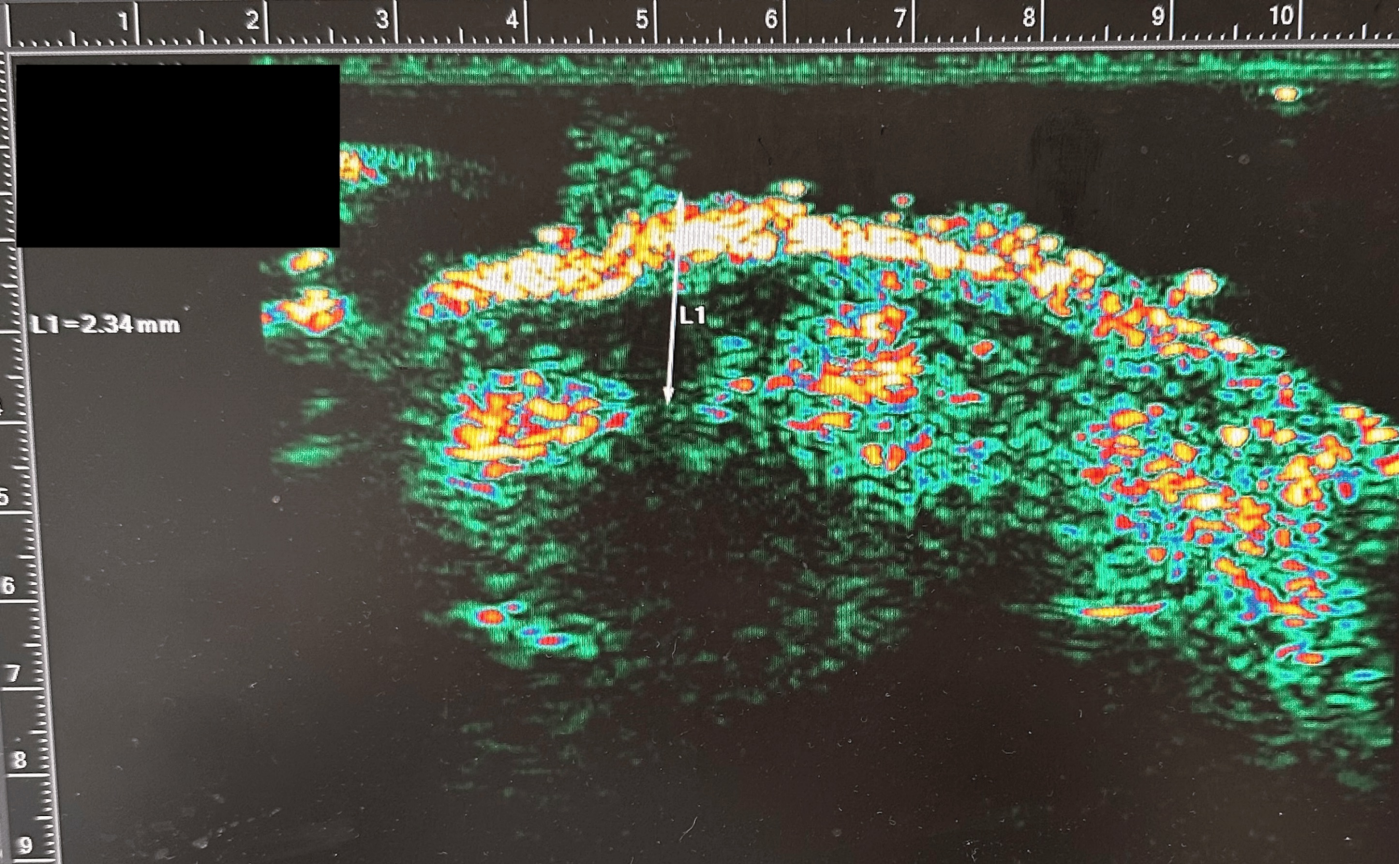
Initial high-resolution dermal ultrasound demonstrating tumor depth prior to treatment. A measured depth of 2.34 mm was within the acceptable treatment threshold of 3 mm. L1 represents the measured depth (2.34 mm) of the tumor prior to radiotherapy treatment.

**Figure 2:**
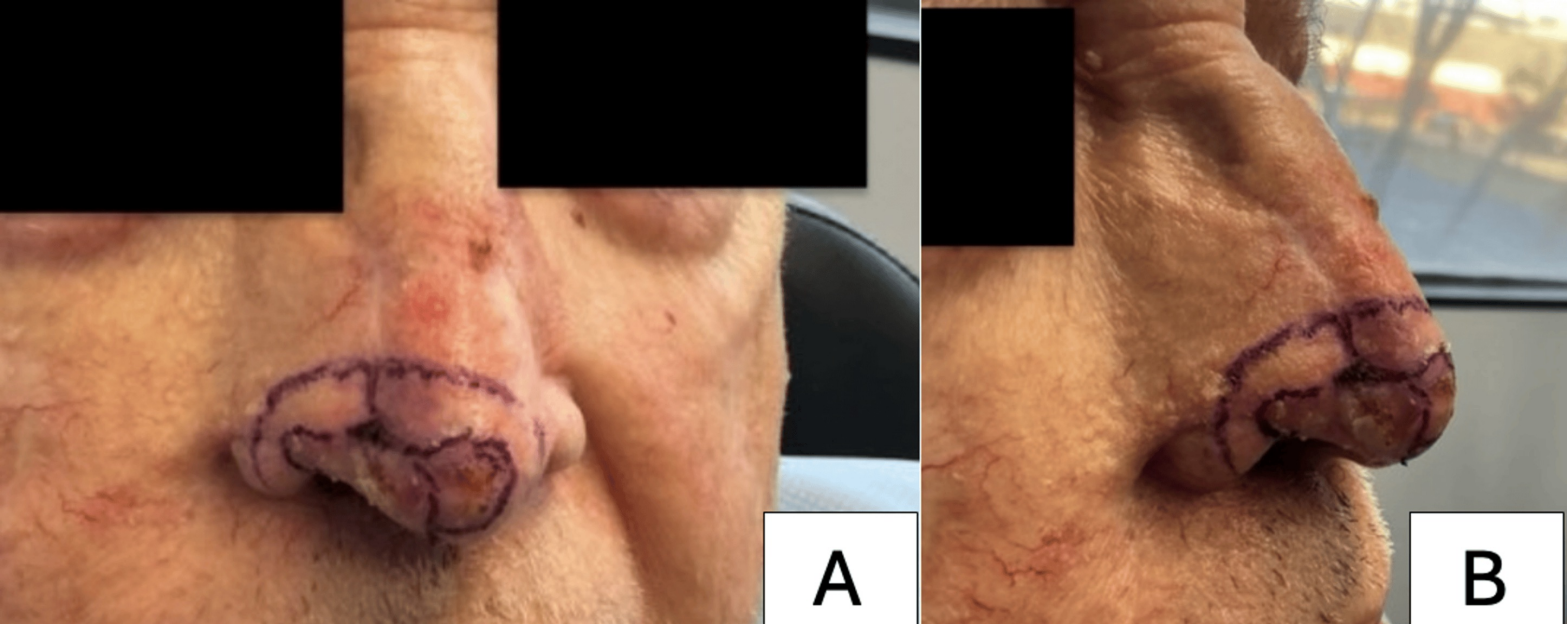
Squamous cell carcinoma in situ involving the right alar rim shown in anterior (A) and lateral (B) views. The initial treatment field measured 3 × 1 cm with a 1 cm margin. The patient provided written and signed consent, allowing publication of this identifiable facial image in an open-access journal.

**Figure 3:**
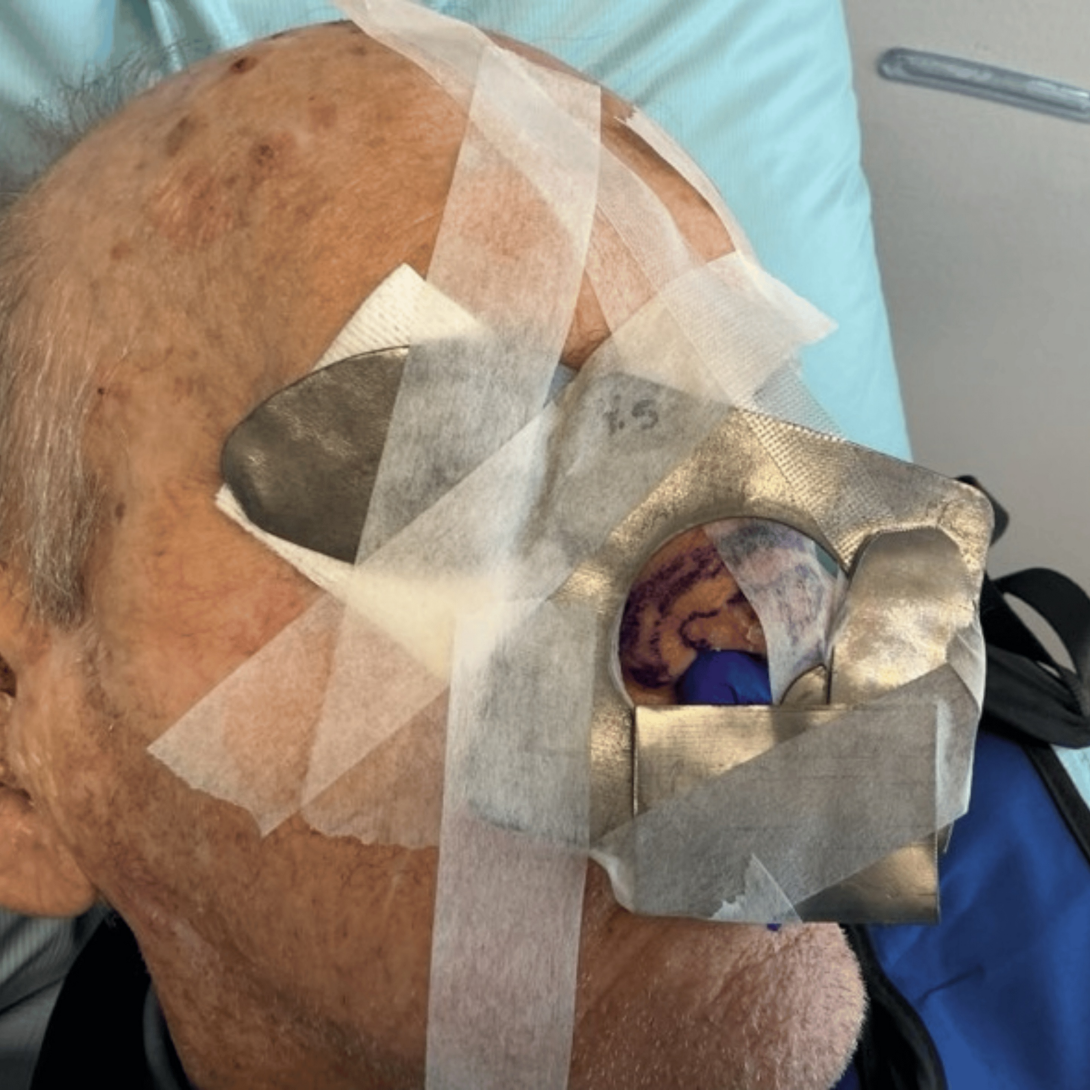
A custom shield and applicator were used during each treatment fraction to protect surrounding healthy tissue and ensure precise radiation delivery to the targeted location. The patient provided written and signed consent, allowing publication of this identifiable facial image in an open-access journal.

At the SRT simulation three weeks later, HRDUS was performed using the SRT-100 Vision system with a 20 MHz transducer, which demonstrated a tumor depth of 2.34 mm (Figure 1). The lesion measured 3 × 1 cm with a treatment margin of 1 cm (Figures 2a-2b). Radiation parameters were as follows: 5 × 3 cm shield (Figure 3), 5 cm applicator, 100 kV energy, and a treatment time of 0.41 minutes per fraction. The patient received 283.31 cGy per fraction for 20 fractions over six weeks (total dose: 5666.20 cGy), with a time, dose, and fractionation factor of 102. A medical physics review was conducted every fifth fraction, including assessment of treatment parameters, quality assurance of dose delivery, and review of patient treatment documentation, ensuring efficacy and continued safe delivery of radiotherapy.

**Figure 4:**
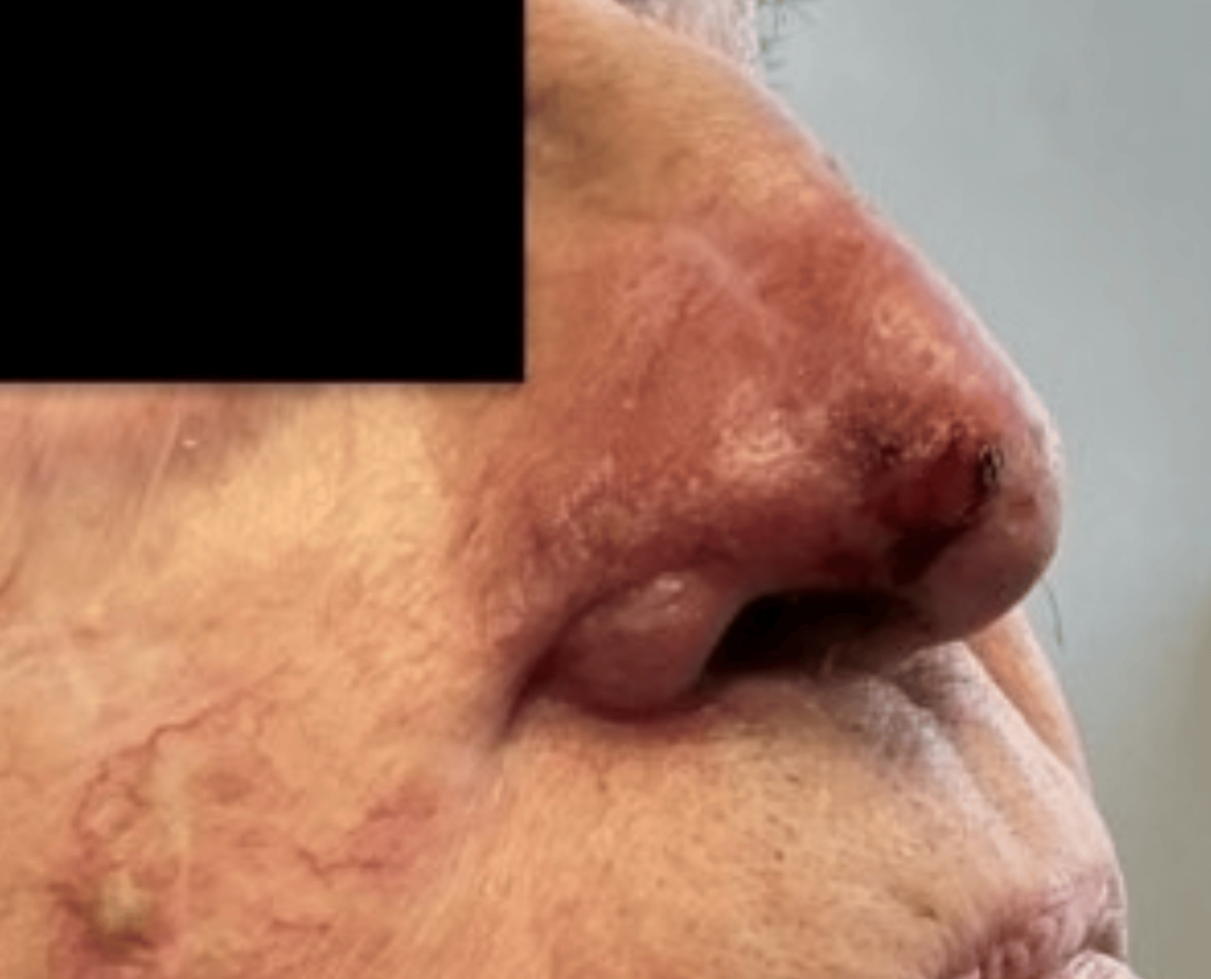
Clinical appearance of the lesion prior to fraction 10. The right alar rim demonstrated erythema, edema, tenderness, and superficial ulceration. These findings were consistent with expected radiation-induced changes and indicated an appropriate therapeutic response. The patient provided written and signed consent, allowing publication of this identifiable facial image in an open-access journal.

The patient reported progressive redness and tenderness over the treatment site. Objectively, there was erythema, tenderness, edema, and superficial ulceration of the right alar rim that was most prominent from fractions 10 to 20 (Figure 4). These findings were consistent with expected radiation-induced changes and indicated an appropriate therapeutic response from the skin cancer. His peak Radiation Therapy Oncology Group (RTOG) toxicity score was 2, corresponding to bright erythema, tenderness, patchy moist desquamation, and moderate edema. The RTOG toxicity scoring system was used to assess acute toxicity on a grading scale from 1 to 4, with grade 4 representing a more severe clinical appearance, such as ulceration, hemorrhage, or necrosis [7].

**Figure 5:**
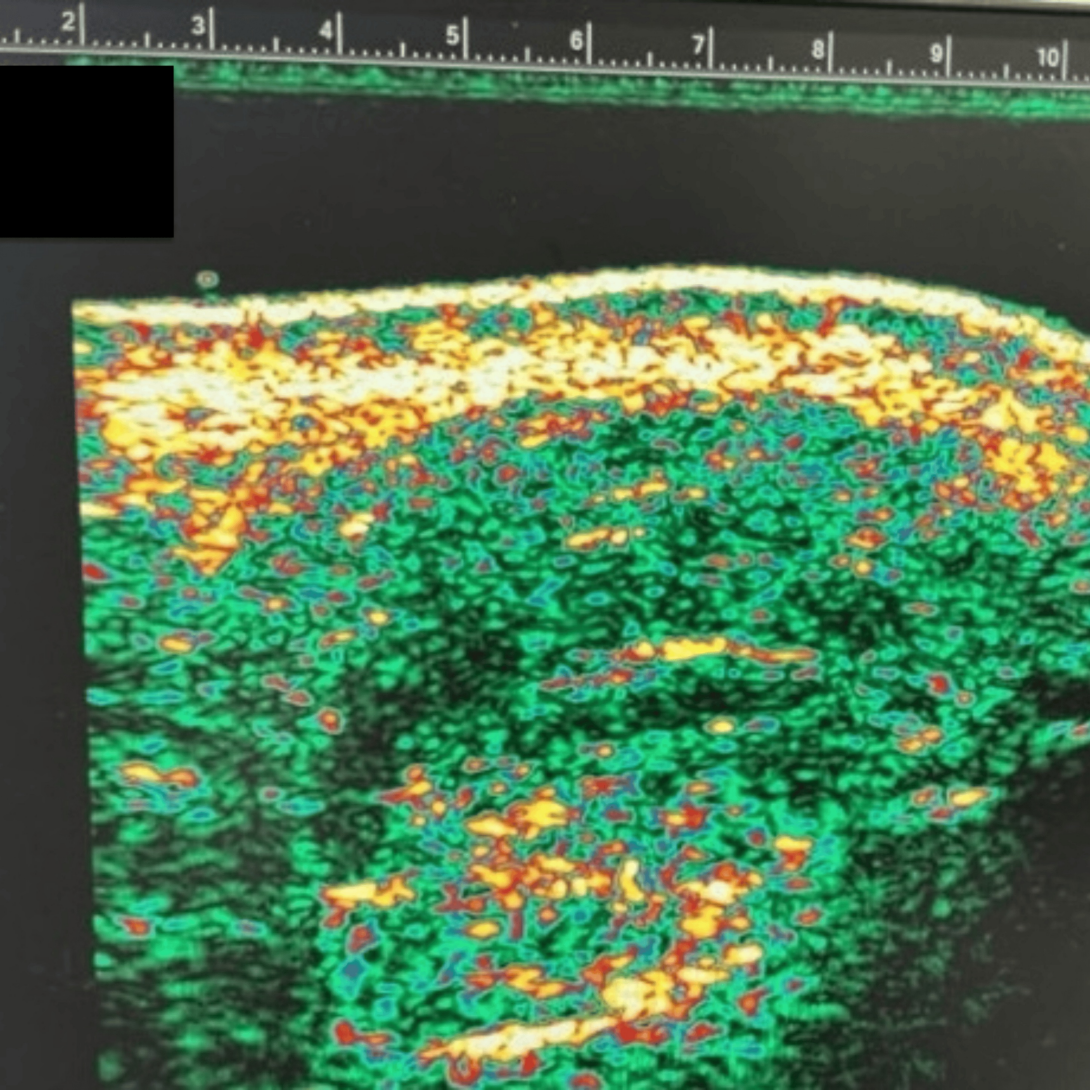
High-resolution dermal ultrasound obtained after completion of 20 treatment fractions over six weeks, with an additional six-week recovery period, demonstrating no detectable residual tumor.

**Figure 6:**
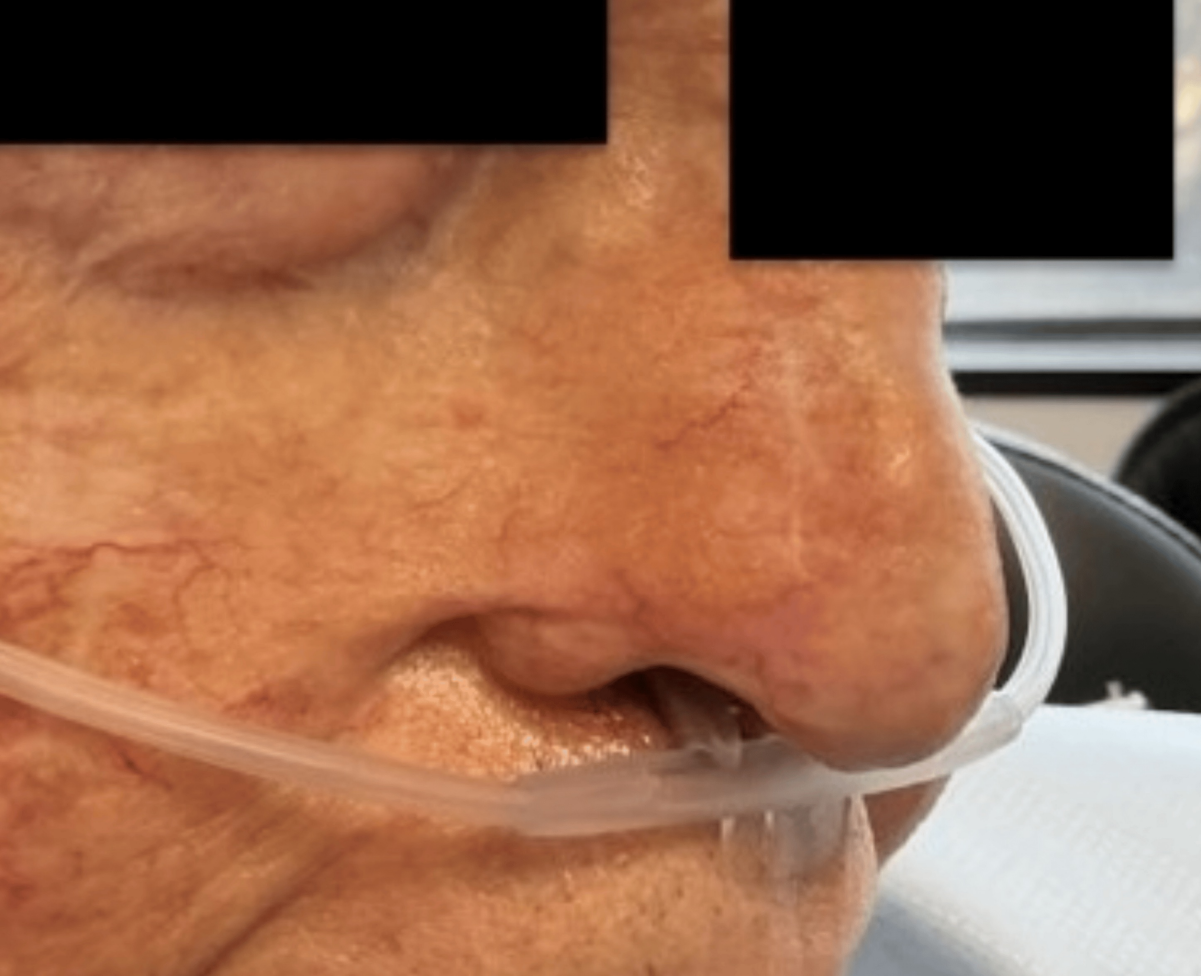
Clinical examination of the right alar rim six weeks after the final treatment fraction demonstrating no clinical evidence of residual skin cancer. The alar contour was preserved with no airway compromise. The RTOG toxicity score was 0. The patient provided written and signed consent, allowing publication of this identifiable facial image in an open-access journal. RTOG: Radiation Therapy Oncology Group

Repeat HRDUS prior to the final fraction demonstrated a tumor depth of 1.42 mm, representing a 0.92 mm reduction from the initial ultrasound measurement. Six weeks after treatment completion, HRDUS demonstrated no detectable residual tumor (Figure 5), and his RTOG toxicity score had resolved to 0 (Figure 6). He tolerated treatment well, was pleased with the cosmetic outcome, and, most importantly, experienced no alar distortion or resultant airway compromise. The patient was discharged to routine dermatologic follow-up for ongoing evaluation and skin surveillance.

## Discussion

Management of NMSC requires individualized treatment selection based on tumor characteristics and patient-specific factors, particularly in medically complex patients. Mohs micrographic surgery is often the preferred treatment for SCCIS in anatomically sensitive areas such as the nasal ala. Utilization of appropriate use criteria (AUC) helps guide decision-making by identifying scenarios in which a tissue-sparing approach provides the greatest benefit and minimizes overuse of this resource-intensive, high-cost procedure for lower-risk cases [8,9].

In this case, an elderly patient with recurrent SCCIS had an AUC score of 7 out of 9, supporting treatment with Mohs surgery. This score was based on several factors, including the recurrent nature of the tumor, its location on the nasal ala within the high-risk “H-zone” of the face, and the diagnosis of SCCIS. According to the AUC, scores of 7 to 9 are considered “appropriate” for Mohs surgery because tissue preservation and complete margin assessment are particularly important in anatomically and cosmetically sensitive locations. However, given his significant comorbidities, a surgical approach was less favorable, and he elected to undergo IG-SRT with HRDUS. Several factors favored a nonsurgical approach, including advanced age, limited mobility, chronic immunosuppression, and the anatomically sensitive location of the tumor. His impaired mobility may have limited his ability to perform postoperative wound care independently, while chronic immunosuppressive therapy may delay wound healing and prolong recovery. Additionally, surgery involving the nasal ala carries potential cosmetic and functional risks, including distortion of the alar rim or airway compromise. Overall, these considerations supported the decision to pursue a noninvasive treatment strategy.

Other nonsurgical treatment modalities were considered and discussed, including PDT, topical pharmacotherapy, and conventional SRT. ED&C was not favored because of the recurrent nature of the lesion, its location on the anatomically and cosmetically sensitive nasal ala, and the absence of histologic margin assessment, which may be particularly important in recurrent tumors. EBX may also be used for superficial cutaneous malignancies in cosmetically sensitive locations; however, unlike IG-SRT, it does not incorporate HRDUS for real-time assessment of tumor depth and treatment response. The ability of IG-SRT to provide image-guided treatment planning and serial monitoring of tumor regression was considered advantageous in this medically complex patient. IG-SRT has demonstrated high efficacy in the treatment of early-stage NMSC, with reported cure rates of ≥99%, whereas PDT has demonstrated lower clearance rates of approximately 77.9%, with limited evidence regarding its effectiveness for recurrent SCCIS [5,10]. An additional limitation of PDT is reduced efficacy in thicker or deeper lesions because of limited tissue penetration. Topical therapies may also be considered in select patients but are generally more dependent on adherence over a prolonged treatment course. Reported clearance rates for SCCIS are lower and more variable, with imiquimod ranging from 73% to 83% and 5-fluorouracil from 27% to 85% [11].

Advantages of IG-SRT include reduced surgical risk, elimination of postoperative wound care, and precise radiation delivery with a favorable safety profile. In the present case, treatment-related toxicity peaked at RTOG grade 2, corresponding to bright erythema, tenderness, patchy moist desquamation, and moderate edema [7]. Importantly, these effects were expected, self-limited, and resolved completely by follow-up, with return to an RTOG score of 0. This clinical course supports the generally favorable tolerability profile of IG-SRT when appropriately administered. A key limitation is the prolonged treatment course, requiring multiple visits per week.

Although Mohs surgery remains the gold standard for cutaneous malignancies of the head and neck, IG-SRT represents a viable alternative in appropriately selected patients and has demonstrated improved outcomes compared with non-image-guided SRT [12,13]. A comparative analysis by Yu et al. demonstrated a statistically significant improvement in local control, defined as complete resolution of a treated lesion without evidence of recurrence, relative to larger high-quality studies utilizing non-image-guided radiotherapy [14].

The key advantage of IG-SRT is the incorporation of HRDUS, which allows for accurate pre-treatment measurement of tumor depth and breadth to guide initial radiation planning [15]. Unlike conventional SRT, HRDUS can also be repeated throughout the treatment course to monitor tumor regression and assess treatment response. This ability to both characterize the lesion before therapy and track response during therapy allows for more individualized dosing while minimizing unnecessary radiation exposure to surrounding healthy tissue. Enhanced diagnostic accuracy and serial assessment of tumor depth may contribute to the favorable local control rates reported with IG-SRT [5]. In this case, HRDUS documented a reduction in tumor depth from an initial depth of 2.34 mm to 1.42 mm prior to the final fraction, and no detectable residual tumor was identified on ultrasound imaging six weeks after treatment completion. However, these findings should be interpreted as evidence of an early clinical and radiographic response rather than definitive long-term cure, as histopathologic confirmation of clearance was not obtained and extended follow-up was unavailable. Post-treatment biopsy was not performed in this case, as management was guided by clinical assessment and serial ultrasound evaluation. Radiation-induced tissue changes may also complicate histopathologic interpretation; therefore, clinical and imaging-based follow-up is often used in this context to assess treatment response [16].

Despite favorable findings of IG-SRT, high-quality evidence remains limited, as no randomized controlled trials have been published [17]. Additional studies, including larger retrospective studies, are needed to further evaluate outcomes. This case report represents a single patient, which limits generalizability. Additionally, although post-treatment ultrasound imaging demonstrated no detectable residual tumor at six weeks, this follow-up interval was relatively short and does not exclude the possibility of future recurrence. Long-term surveillance was not available because subsequent follow-up care was transitioned to the patient’s general dermatologist. Furthermore, while HRDUS was used to monitor treatment response, histopathologic confirmation of complete tumor clearance was not obtained. Therefore, the observed findings should be interpreted as evidence of short-term clinical and radiographic resolution rather than definitive cure. Further studies with larger sample sizes and longer follow-up are needed to better define the role of IG-SRT in the management of early-stage NMSCs.

## Conclusions

This case highlights IG-SRT as a feasible, tissue-sparing treatment option for recurrent SCCIS of the nasal ala in a medically complex patient who was a poor surgical candidate. The integration of HRDUS allowed for precise tumor measurement, monitoring of treatment response, and individualized radiation delivery, resulting in clinical and sonographic resolution with favorable cosmetic and functional outcomes. Although Mohs micrographic surgery remains the standard of care for cutaneous malignancies in cosmetically sensitive areas, IG-SRT may represent a reasonable alternative for appropriately selected patients in whom surgery is contraindicated or less desirable. Importantly, the observed findings reflect short-term clinical and ultrasound-based response, as histopathologic confirmation of clearance was not obtained, and long-term follow-up was unavailable. This case emphasizes the importance of individualized treatment selection in elderly or medically complex patients with NMSC. Further studies with larger cohorts and longer follow-up are warranted to better define its long-term efficacy and role in the management of NMSC.
